# *trans*-Bis[(2-bromo­phen­yl)diphenyl­phosphane-κ*P*]carbonyl­chlorido­rhodium(I)

**DOI:** 10.1107/S1600536812012421

**Published:** 2012-03-31

**Authors:** Frederick P. Malan, Rehana Malgas-Enus, Reinout Meijboom

**Affiliations:** a Research Centre for Synthesis and Catalysis, Department of Chemistry, University of Johannesburg, PO Box 524, Auckland Park 2006, Johannesburg, South Africa

## Abstract

The title compound, *trans*-[RhCl(C_18_H_14_BrP)_2_(CO)], has a slightly disordered square-planar geometry with the Rh ion^I^ situated on an inversion the centre and carbon­yl–chloride disorder observed as a result of the crystallographic inversion symmetry. Selected geometric parameters include: Rh—P = 2.3430 (8) Å, Rh—Cl = 2.434 (3) Å, Rh—C = 1.722 (8) Å, P—Rh—P = 180.00 (3)°, P—Rh—Cl = 95.40 (7)°, 84.60 (7)° and Rh—C—O = 177.9 (8)°.

## Related literature

For background to Vaska-type complexes, see: Roodt *et al.* (2003 ▶); Lamb *et al.* (2009 ▶); Vaska & Di Luzio (1961 ▶). For related complexes, see: Burgoyne *et al.* (2010 ▶); Makhoba *et al.* (2011 ▶); Meijboom (2011 ▶); Meijboom *et al.* (2004 ▶); Otto *et al.* (2000 ▶); Otto & Roodt (2004 ▶); Chen *et al.* (1991 ▶); Kemp *et al.* (1995 ▶). 
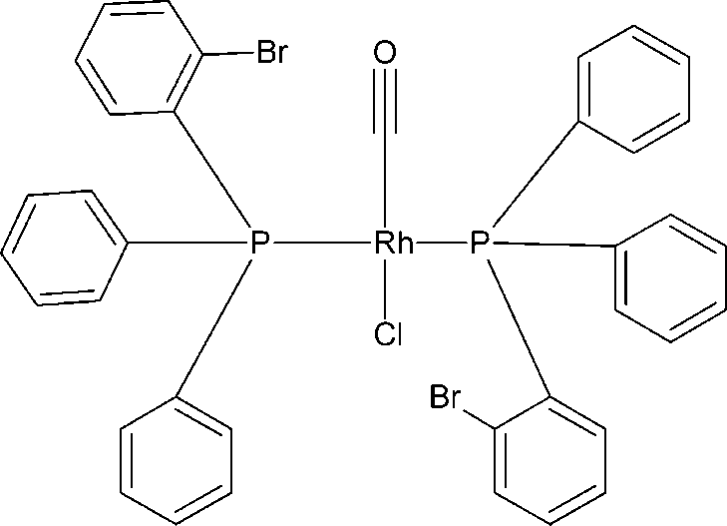


## Experimental

### 

#### Crystal data

[RhCl(C_18_H_14_BrP)_2_(CO)]
*M* *_r_* = 848.71
Monoclinic, 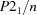

*a* = 9.3250 (5) Å
*b* = 17.041 (1) Å
*c* = 10.8880 (6) Å
β = 111.229 (1)°
*V* = 1612.77 (16) Å^3^
*Z* = 2
Mo *K*α radiation
μ = 3.22 mm^−1^
*T* = 100 K
0.23 × 0.13 × 0.11 mm

#### Data collection

Bruker APEXII CCD diffractometer
Absorption correction: multi-scan (*SADABS*; Bruker, 2005 ▶) *T*_min_ = 0.618, *T*_max_ = 0.697
15235 measured reflections
4032 independent reflections
3531 reflections with *I* > 2σ(*I*)
*R*_int_ = 0.026

#### Refinement

*R*[*F* ^2^ > 2σ(*F* ^2^)] = 0.027
*wR*(*F* ^2^) = 0.063
*S* = 1.11
4032 reflections
214 parameters
H-atom parameters constrained
Δρ_max_ = 0.67 e Å^−3^
Δρ_min_ = −0.81 e Å^−3^

### 

Data collection: *APEX2* (Bruker, 2005 ▶); cell refinement: *SAINT* (Bruker, 2004 ▶); data reduction: *SAINT*; program(s) used to solve structure: *SHELXS97* (Sheldrick, 2008 ▶); program(s) used to refine structure: *SHELXL97* (Sheldrick, 2008 ▶); molecular graphics: *SHELXTL* (Sheldrick, 2008 ▶) and *DIAMOND* (Brandenburg & Putz (2005 ▶); software used to prepare material for publication: *SHELXTL*.

## Supplementary Material

Crystal structure: contains datablock(s) global, I. DOI: 10.1107/S1600536812012421/hp2033sup1.cif

Structure factors: contains datablock(s) I. DOI: 10.1107/S1600536812012421/hp2033Isup2.hkl

Additional supplementary materials:  crystallographic information; 3D view; checkCIF report

## Figures and Tables

**Fig. 1.**
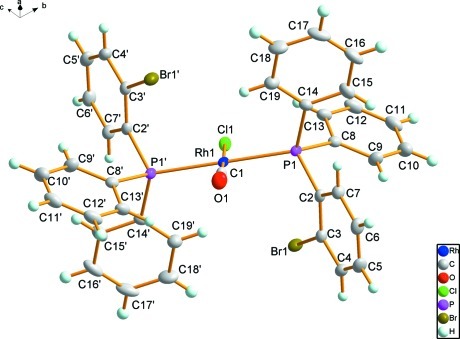
The structure of the title compound, showing 50% probability displacement ellipsoids. All rings have been numbered in the same, systematic manner. H atoms are depicted by arbitrary size spheres. Hashed atoms are generated by symmetry (-x + 1, -y, -z + 2).

## supplementary crystallographic information

### Comment

A vast range of different Vaska-type complexes have been synthesized, reported and spectroscopically studied (Roodt *et al.*, 2003; Lamb *et al.*; 2009) since the synthesis and correct formulation of the original Vaska complex, *trans*-[Ir(CO)Cl(C_18_H_15_P)_2_], by Vaska & Di Luzio (1961). This class of symmetrical square-planar complexes (including Rh, Ir, Pd and Pt) usually crystallizes with the metal atom on a crystallographic inversion centre, resulting in a disordered packing arrangement (Chen *et al.*, 1991; Otto *et al.*, 2000; Otto & Roodt, 2004; Meijboom *et al.*, 2004). The title compound serves as yet another complex to add to the Vaska's complex range with varying Group 5 ligand systems possessing different stereoelectronic properties.

In the title compound, the Rh atom lies at the centre of a slightly distorted square-planar geometric arrangement. The Rh atom crystallizes on a centre of symmetry, a crystallographic inversion centre, and has the carbonyl and chloro- ligands disordered at a 0.5:0.5 ratio. The stereoelectronic property of the phosphane with the bromo-functionality is indicated by the smaller O1—Rh1—P1 angle (85.39 (19)°), which is translated through symmetry to the inverted side of the molecule.

Selected spectroscopic data of the current compound is comparable to other similar complexes reported previously by Roodt *et al.* (2003) and Otto & Roodt (2004). However, the interesting difference in the magnitude of v(CO) for the solid and solution (in DCM) states of the title compound is ascribed to the packing of the molecules, which slightly distorts the Rh C—O angle. This effect was previously observed and reported for a polymorph of *trans*-[Rh(CO)Cl{PPh_3_}_2_] (Kemp *et al.*, 1995).

### Experimental

A solution of dichlorotetracarbonyldirhodium (0.050 g, 0.13 mmol) in acetone (3 cm^3^) was slowly added to a solution of the phosphane, C_18_H_14_BrP (0.176 g, 0.51 mmol) in acetone (3 cm^3^) at room temperature,after which the mixture was left to crystallize. Slow evaporation of the solvent afforded the title compound as yellow crystals. Spectroscopic analysis: ^31^P{H} NMR (CDCl_3_, 162.0 MHz, p.p.m.): 33.1[d, ^1^J(Rh—P) = 132.8 Hz, 2P]; IR ν(CO): 1950.8 cm^-1^; (CD_2_Cl_2_) ν(CO): 1973.2 cm^-1^.

### Refinement

The aromatic H atoms were placed in geometrically idealized positions (C—H = 0.93 Å) and constrained to ride on their parent atoms, with *U*_iso_(H) = 1.2*U*_eq_(C) for all H atoms. The highest calculated residual electron density is 0.66 e.Å^-3^ at 1.597 Å from C15, which bears no physical meaning.

### Crystal data

**Table:**

[RhCl(C_18_H_14_BrP)_2_(CO)]	*F*(000) = 840
*M**_r_* = 848.71	*D*_x_ = 1.748 Mg m^−^^3^
Monoclinic, *P*2_1_/*n*	Mo *K*α radiation, λ = 0.71069 Å
Hall symbol: -P 2yn	Cell parameters from 6197 reflections
*a* = 9.3250 (5) Å	θ = 2.4–28.3°
*b* = 17.041 (1) Å	µ = 3.22 mm^−^^1^
*c* = 10.8880 (6) Å	*T* = 100 K
β = 111.229 (1)°	Rectangular, yellow
*V* = 1612.77 (16) Å^3^	0.23 × 0.13 × 0.11 mm
*Z* = 2	

### Data collection

**Table:**

Bruker APEXII CCD diffractometer	3531 reflections with *I* > 2σ(*I*)
Graphite monochromator	*R*_int_ = 0.026
φ and ω scans	θ_max_ = 28.4°, θ_min_ = 2.3°
Absorption correction: multi-scan (*SADABS*; Bruker, 2005)	*h* = −12→12
*T*_min_ = 0.618, *T*_max_ = 0.697	*k* = −22→22
15235 measured reflections	*l* = −14→14
4032 independent reflections	

### Refinement

**Table:**

Refinement on *F*^2^	Primary atom site location: structure-invariant direct methods
Least-squares matrix: full	Secondary atom site location: difference Fourier map
*R*[*F*^2^ > 2σ(*F*^2^)] = 0.027	Hydrogen site location: inferred from neighbouring sites
*wR*(*F*^2^) = 0.063	H-atom parameters constrained
*S* = 1.11	*w* = 1/[σ^2^(*F*_o_^2^) + (0.0246*P*)^2^ + 1.6443*P*] where *P* = (*F*_o_^2^ + 2*F*_c_^2^)/3
4032 reflections	(Δ/σ)_max_ = 0.001
214 parameters	Δρ_max_ = 0.67 e Å^−^^3^
0 restraints	Δρ_min_ = −0.81 e Å^−^^3^

### Special details

**Table:**

Geometry. All e.s.d.'s (except the e.s.d. in the dihedral angle between two l.s. planes) are estimated using the full covariance matrix. The cell e.s.d.'s are taken into account individually in the estimation of e.s.d.'s in distances, angles and torsion angles; correlations between e.s.d.'s in cell parameters are only used when they are defined by crystal symmetry. An approximate (isotropic) treatment of cell e.s.d.'s is used for estimating e.s.d.'s involving l.s. planes.
Refinement. Refinement of *F*^2^ against ALL reflections. The weighted *R*-factor *wR* and goodness of fit *S* are based on *F*^2^, conventional *R*-factors *R* are based on *F*, with *F* set to zero for negative *F*^2^. The threshold expression of *F*^2^ > σ(*F*^2^) is used only for calculating *R*-factors(gt) *etc*. and is not relevant to the choice of reflections for refinement. *R*-factors based on *F*^2^ are statistically about twice as large as those based on *F*, and *R*- factors based on ALL data will be even larger.

### Fractional atomic coordinates and isotropic or equivalent isotropic displacement parameters (Å^2^)

**Table:**

	*x*	*y*	*z*	*U*_iso_*/*U*_eq_	Occ. (<1)
Rh1	0.5	0	1	0.01378 (7)	
C1	0.5773 (7)	−0.0478 (4)	0.8993 (6)	0.0178 (11)	0.5
O1	0.6340 (10)	−0.0800 (5)	0.8343 (9)	0.0236 (17)	0.5
Cl1	0.6037 (3)	−0.06666 (16)	0.8527 (3)	0.0174 (4)	0.5
P1	0.50550 (6)	0.11189 (4)	0.87674 (5)	0.01442 (12)	
Br1	0.13942 (3)	0.061590 (16)	0.75242 (2)	0.02135 (7)	
C7	0.4960 (3)	0.09681 (15)	0.6146 (2)	0.0189 (5)	
H7	0.6006	0.1083	0.6484	0.023*	
C2	0.4152 (3)	0.09234 (14)	0.7000 (2)	0.0156 (4)	
C8	0.4165 (3)	0.20586 (14)	0.8906 (2)	0.0164 (5)	
C12	0.3697 (3)	0.30670 (16)	1.0277 (3)	0.0269 (6)	
H12	0.379	0.3248	1.1108	0.032*	
C14	0.7017 (3)	0.14283 (14)	0.9010 (2)	0.0174 (5)	
C19	0.8252 (3)	0.09824 (16)	0.9810 (2)	0.0237 (5)	
H19	0.8069	0.0527	1.02	0.028*	
C15	0.7317 (3)	0.21089 (16)	0.8434 (3)	0.0255 (5)	
H15	0.6504	0.2418	0.7908	0.031*	
C18	0.9756 (3)	0.12109 (19)	1.0032 (3)	0.0314 (6)	
H18	1.0574	0.0914	1.0582	0.038*	
C11	0.2947 (3)	0.35167 (16)	0.9183 (3)	0.0263 (6)	
H11	0.2529	0.3999	0.9275	0.032*	
C4	0.1846 (3)	0.06291 (15)	0.5096 (2)	0.0215 (5)	
H4	0.0797	0.0523	0.475	0.026*	
C6	0.4238 (3)	0.08449 (16)	0.4807 (2)	0.0231 (5)	
H6	0.4803	0.0869	0.4259	0.028*	
C17	1.0033 (3)	0.18774 (18)	0.9436 (3)	0.0326 (7)	
H17	1.1039	0.2025	0.957	0.039*	
C9	0.3404 (3)	0.25267 (16)	0.7805 (2)	0.0233 (5)	
H9	0.3292	0.2348	0.6968	0.028*	
C3	0.2589 (3)	0.07322 (14)	0.6440 (2)	0.0169 (5)	
C5	0.2675 (3)	0.06855 (15)	0.4277 (2)	0.0234 (5)	
H5	0.2187	0.0617	0.3374	0.028*	
C13	0.4314 (3)	0.23448 (15)	1.0149 (2)	0.0220 (5)	
H13	0.4831	0.2049	1.0897	0.026*	
C10	0.2816 (3)	0.32529 (16)	0.7949 (3)	0.0274 (6)	
H10	0.2332	0.3563	0.7213	0.033*	
C16	0.8815 (3)	0.23287 (17)	0.8639 (3)	0.0306 (6)	
H16	0.9005	0.278	0.8241	0.037*	

### Atomic displacement parameters (Å^2^)

**Table:**

	*U*^11^	*U*^22^	*U*^33^	*U*^12^	*U*^13^	*U*^23^
Rh1	0.01221 (11)	0.01690 (13)	0.01105 (11)	−0.00151 (9)	0.00278 (9)	0.00207 (10)
C1	0.021 (3)	0.017 (3)	0.015 (3)	0.000 (2)	0.007 (2)	0.004 (2)
O1	0.024 (4)	0.027 (4)	0.026 (3)	0.008 (2)	0.017 (2)	0.001 (2)
Cl1	0.0204 (12)	0.0177 (11)	0.0171 (11)	0.0033 (8)	0.0105 (7)	0.0011 (8)
P1	0.0130 (3)	0.0175 (3)	0.0124 (3)	−0.0011 (2)	0.0041 (2)	0.0022 (2)
Br1	0.01558 (11)	0.03052 (15)	0.01759 (12)	−0.00259 (9)	0.00558 (9)	0.00252 (10)
C7	0.0188 (11)	0.0208 (12)	0.0185 (11)	0.0022 (9)	0.0085 (9)	0.0024 (9)
C2	0.0193 (11)	0.0150 (11)	0.0129 (10)	0.0016 (9)	0.0063 (9)	0.0014 (9)
C8	0.0139 (10)	0.0191 (12)	0.0164 (11)	−0.0030 (9)	0.0059 (9)	−0.0013 (9)
C12	0.0326 (14)	0.0271 (14)	0.0274 (13)	−0.0124 (11)	0.0187 (12)	−0.0121 (11)
C14	0.0161 (10)	0.0194 (12)	0.0178 (11)	−0.0036 (9)	0.0074 (9)	−0.0034 (9)
C19	0.0190 (11)	0.0281 (14)	0.0228 (12)	−0.0024 (10)	0.0062 (10)	0.0010 (11)
C15	0.0279 (13)	0.0192 (13)	0.0335 (14)	−0.0036 (10)	0.0160 (11)	−0.0032 (11)
C18	0.0156 (12)	0.0410 (17)	0.0350 (15)	−0.0016 (11)	0.0059 (11)	−0.0044 (13)
C11	0.0250 (13)	0.0190 (13)	0.0393 (15)	−0.0041 (10)	0.0171 (12)	−0.0081 (11)
C4	0.0219 (12)	0.0214 (13)	0.0171 (11)	−0.0012 (10)	0.0022 (9)	0.0012 (10)
C6	0.0304 (13)	0.0250 (14)	0.0176 (12)	0.0037 (11)	0.0132 (10)	0.0024 (10)
C17	0.0219 (13)	0.0383 (17)	0.0443 (17)	−0.0140 (12)	0.0201 (12)	−0.0196 (14)
C9	0.0255 (12)	0.0235 (13)	0.0178 (11)	0.0023 (10)	0.0041 (10)	−0.0032 (10)
C3	0.0195 (11)	0.0169 (12)	0.0151 (11)	0.0016 (9)	0.0070 (9)	0.0023 (9)
C5	0.0320 (14)	0.0247 (14)	0.0122 (11)	0.0004 (11)	0.0064 (10)	−0.0012 (10)
C13	0.0243 (12)	0.0239 (13)	0.0171 (11)	−0.0075 (10)	0.0067 (10)	−0.0039 (10)
C10	0.0265 (13)	0.0207 (13)	0.0294 (14)	0.0011 (11)	0.0033 (11)	−0.0015 (11)
C16	0.0345 (14)	0.0236 (14)	0.0440 (17)	−0.0130 (12)	0.0266 (13)	−0.0107 (12)

### Geometric parameters (Å, º)

**Table:**

Rh1—C1^i^	1.720 (7)	C19—C18	1.390 (3)
Rh1—C1	1.720 (7)	C19—H19	0.93
Rh1—P1	2.3429 (6)	C15—C16	1.384 (4)
Rh1—P1^i^	2.3429 (6)	C15—H15	0.93
Rh1—Cl1^i^	2.433 (3)	C18—C17	1.378 (4)
Rh1—Cl1	2.433 (3)	C18—H18	0.93
C1—O1	1.163 (7)	C11—C10	1.379 (4)
P1—C14	1.829 (2)	C11—H11	0.93
P1—C2	1.831 (2)	C4—C5	1.379 (4)
P1—C8	1.835 (2)	C4—C3	1.385 (3)
Br1—C3	1.904 (2)	C4—H4	0.93
C7—C6	1.383 (3)	C6—C5	1.386 (4)
C7—C2	1.394 (3)	C6—H6	0.93
C7—H7	0.93	C17—C16	1.386 (4)
C2—C3	1.399 (3)	C17—H17	0.93
C8—C13	1.397 (3)	C9—C10	1.385 (4)
C8—C9	1.400 (3)	C9—H9	0.93
C12—C11	1.375 (4)	C5—H5	0.93
C12—C13	1.387 (4)	C13—H13	0.93
C12—H12	0.93	C10—H10	0.93
C14—C19	1.391 (3)	C16—H16	0.93
C14—C15	1.394 (4)		
			
C1^i^—Rh1—C1	180.0000 (10)	C18—C19—H19	119.7
C1^i^—Rh1—P1	94.63 (19)	C14—C19—H19	119.7
C1—Rh1—P1	85.37 (19)	C16—C15—C14	120.5 (3)
C1^i^—Rh1—P1^i^	85.37 (19)	C16—C15—H15	119.7
C1—Rh1—P1^i^	94.63 (19)	C14—C15—H15	119.7
P1—Rh1—P1^i^	180	C17—C18—C19	119.9 (3)
C1^i^—Rh1—Cl1^i^	1.46 (18)	C17—C18—H18	120.1
C1—Rh1—Cl1^i^	178.54 (19)	C19—C18—H18	120.1
P1—Rh1—Cl1^i^	95.39 (7)	C12—C11—C10	120.0 (2)
P1^i^—Rh1—Cl1^i^	84.61 (7)	C12—C11—H11	120
C1^i^—Rh1—Cl1	178.54 (18)	C10—C11—H11	120
C1—Rh1—Cl1	1.46 (19)	C5—C4—C3	119.4 (2)
P1—Rh1—Cl1	84.61 (7)	C5—C4—H4	120.3
P1^i^—Rh1—Cl1	95.39 (7)	C3—C4—H4	120.3
Cl1^i^—Rh1—Cl1	180.0000 (10)	C7—C6—C5	120.4 (2)
O1—C1—Rh1	177.9 (8)	C7—C6—H6	119.8
C14—P1—C2	105.04 (11)	C5—C6—H6	119.8
C14—P1—C8	101.26 (11)	C18—C17—C16	120.1 (2)
C2—P1—C8	101.29 (10)	C18—C17—H17	120
C14—P1—Rh1	112.34 (8)	C16—C17—H17	120
C2—P1—Rh1	110.92 (8)	C10—C9—C8	120.7 (2)
C8—P1—Rh1	123.96 (8)	C10—C9—H9	119.7
C6—C7—C2	121.3 (2)	C8—C9—H9	119.7
C6—C7—H7	119.3	C4—C3—C2	122.2 (2)
C2—C7—H7	119.3	C4—C3—Br1	117.41 (18)
C7—C2—C3	116.8 (2)	C2—C3—Br1	120.34 (17)
C7—C2—P1	122.46 (18)	C4—C5—C6	119.7 (2)
C3—C2—P1	120.68 (17)	C4—C5—H5	120.1
C13—C8—C9	118.2 (2)	C6—C5—H5	120.1
C13—C8—P1	119.67 (19)	C12—C13—C8	120.5 (2)
C9—C8—P1	122.05 (18)	C12—C13—H13	119.8
C11—C12—C13	120.5 (2)	C8—C13—H13	119.8
C11—C12—H12	119.8	C11—C10—C9	120.2 (3)
C13—C12—H12	119.8	C11—C10—H10	119.9
C19—C14—C15	118.7 (2)	C9—C10—H10	119.9
C19—C14—P1	119.39 (19)	C15—C16—C17	120.1 (3)
C15—C14—P1	121.86 (19)	C15—C16—H16	120
C18—C19—C14	120.7 (3)	C17—C16—H16	120
			
C1^i^—Rh1—C1—O1	9E1 (10)	Rh1—P1—C8—C9	−141.23 (18)
P1—Rh1—C1—O1	10E1 (2)	C2—P1—C14—C19	−117.8 (2)
P1^i^—Rh1—C1—O1	−8E1 (2)	C8—P1—C14—C19	137.1 (2)
Cl1^i^—Rh1—C1—O1	−2E1 (3)	Rh1—P1—C14—C19	2.8 (2)
Cl1—Rh1—C1—O1	16E1 (3)	C2—P1—C14—C15	63.4 (2)
C1^i^—Rh1—P1—C14	113.6 (2)	C8—P1—C14—C15	−41.7 (2)
C1—Rh1—P1—C14	−66.4 (2)	Rh1—P1—C14—C15	−175.97 (18)
P1^i^—Rh1—P1—C14	−93.80 (10)	C15—C14—C19—C18	0.1 (4)
Cl1^i^—Rh1—P1—C14	112.34 (10)	P1—C14—C19—C18	−178.8 (2)
Cl1—Rh1—P1—C14	−67.66 (10)	C19—C14—C15—C16	1.0 (4)
C1^i^—Rh1—P1—C2	−129.2 (2)	P1—C14—C15—C16	179.8 (2)
C1—Rh1—P1—C2	50.8 (2)	C14—C19—C18—C17	−1.3 (4)
P1^i^—Rh1—P1—C2	23.42 (13)	C13—C12—C11—C10	−0.5 (4)
Cl1^i^—Rh1—P1—C2	−130.44 (10)	C2—C7—C6—C5	1.1 (4)
Cl1—Rh1—P1—C2	49.56 (10)	C19—C18—C17—C16	1.4 (4)
C1^i^—Rh1—P1—C8	−8.5 (2)	C13—C8—C9—C10	0.0 (4)
C1—Rh1—P1—C8	171.5 (2)	P1—C8—C9—C10	−176.5 (2)
P1^i^—Rh1—P1—C8	144.10 (13)	C5—C4—C3—C2	2.6 (4)
Cl1^i^—Rh1—P1—C8	−9.76 (11)	C5—C4—C3—Br1	−178.16 (19)
Cl1—Rh1—P1—C8	170.24 (11)	C7—C2—C3—C4	−3.1 (4)
C6—C7—C2—C3	1.3 (4)	P1—C2—C3—C4	175.85 (19)
C6—C7—C2—P1	−177.7 (2)	C7—C2—C3—Br1	177.59 (18)
C14—P1—C2—C7	4.1 (2)	P1—C2—C3—Br1	−3.4 (3)
C8—P1—C2—C7	109.2 (2)	C3—C4—C5—C6	−0.1 (4)
Rh1—P1—C2—C7	−117.49 (19)	C7—C6—C5—C4	−1.7 (4)
C14—P1—C2—C3	−174.83 (19)	C11—C12—C13—C8	−0.9 (4)
C8—P1—C2—C3	−69.8 (2)	C9—C8—C13—C12	1.2 (4)
Rh1—P1—C2—C3	63.6 (2)	P1—C8—C13—C12	177.67 (19)
C14—P1—C8—C13	−84.6 (2)	C12—C11—C10—C9	1.6 (4)
C2—P1—C8—C13	167.38 (19)	C8—C9—C10—C11	−1.3 (4)
Rh1—P1—C8—C13	42.4 (2)	C14—C15—C16—C17	−0.9 (4)
C14—P1—C8—C9	91.8 (2)	C18—C17—C16—C15	−0.3 (4)
C2—P1—C8—C9	−16.2 (2)		

Symmetry code: (i) −*x*+1, −*y*, −*z*+2.
